# A Systems Biology Approach to the Characterization of Stress Response in *Dermacentor reticulatus* Tick Unfed Larvae

**DOI:** 10.1371/journal.pone.0089564

**Published:** 2014-02-21

**Authors:** Margarita Villar, Marina Popara, Nieves Ayllón, Isabel G. Fernández de Mera, Lourdes Mateos-Hernández, Ruth C. Galindo, Marina Manrique, Raquel Tobes, José de la Fuente

**Affiliations:** 1 SaBio. Instituto de Investigación en Recursos Cinegéticos IREC-CSIC-UCLM-JCCM, Ciudad Real, Spain; 2 Department of Veterinary Pathobiology, Center for Veterinary Health Sciences, Oklahoma State University, Stillwater, Oklahoma, United States of America; 3 Oh no sequences! Research group, Era7 Bioinformatics, Granada, Spain; Washington State University, United States of America

## Abstract

**Background:**

*Dermacentor reticulatus* (Fabricius, 1794) is distributed in Europe and Asia where it infests and transmits disease-causing pathogens to humans, pets and other domestic and wild animals. However, despite its role as a vector of emerging or re-emerging diseases, very little information is available on the genome, transcriptome and proteome of *D. reticulatus*. Tick larvae are the first developmental stage to infest hosts, acquire infection and transmit pathogens that are transovarially transmitted and are exposed to extremely stressing conditions. In this study, we used a systems biology approach to get an insight into the mechanisms active in *D. reticulatus* unfed larvae, with special emphasis on stress response.

**Principal Findings:**

The results support the use of paired end RNA sequencing and proteomics informed by transcriptomics (PIT) for the analysis of transcriptomics and proteomics data, particularly for organisms such as *D. reticulatus* with little sequence information available. The results showed that metabolic and cellular processes involved in protein synthesis were the most active in *D. reticulatus* unfed larvae, suggesting that ticks are very active during this life stage. The stress response was activated in *D. reticulatus* unfed larvae and a *Rickettsia* sp. similar to *R. raoultii* was identified in these ticks.

**Significance:**

The activation of stress responses in *D. reticulatus* unfed larvae likely counteracts the negative effect of temperature and other stress conditions such as *Rickettsia* infection and favors tick adaptation to environmental conditions to increase tick survival. These results show mechanisms that have evolved in *D. reticulatus* ticks to survive under stress conditions and suggest that these mechanisms are conserved across hard tick species. Targeting some of these proteins by vaccination may increase tick susceptibility to natural stress conditions, which in turn reduce tick survival and reproduction, thus reducing tick populations and vector capacity for tick-borne pathogens.

## Introduction

Ticks are blood-sucking ectoparasites that infest and transmit pathogens to humans and animals. *Dermacentor reticulatus* (Fabricius, 1794) is a three hosts tick (larvae, nymphs and adults feed on different hosts) distributed in Europe and Asia where it infests humans, pets and other domestic and wild animals. *D. reticulatus* transmit disease-causing pathogens such as *Rickettsia slovaca* (tick-borne lymphoadenopathy; TIBOLA), Omsk hemorrhagic fever virus (OHFV; Omsk hemorrhagic fever), tick-borne encephalitis virus (TBEV; tick-borne encephalitis), *Francisella tularensis* (tularemia) and *Babesia canis* (canine babesiosis) [1]–[3].

Despite its role as a vector of emerging or re-emerging diseases, very little information is available on the genome, transcriptome and proteome of *D. reticulatus* (115 nucleotide sequences of which only 15 were not of rRNA and 9 protein sequences deposited in the GenBank on June 2013).

This research focused on tick larvae because this is the first developmental stage to infest hosts, acquire infection and transmit pathogens that are transovarially transmitted. Additionally, *D. reticulatus* larvae hatch at a temperature range of 20–34°C and can survive for 83.5 days at 5°C and 100% relative humidity [4]. However, under natural conditions, larvae are active within 16–20 days after hatching and survive about a month before feeding [5]. *D. reticulatus* larvae feed on small mammals and are active during the summer [6].

All these facts put tick unfed larvae under extremely stressing conditions. For example, under natural conditions only 5–15% *D. reticulatus* larvae produced from a single clutch are activated [5]. In this study, we characterized the transcriptome and proteome of *D. reticulatus* unfed larvae to get an insight into the mechanisms active at this developmental stage, with special emphasis on stress response.

## Results and Discussion

### *D. reticulatus* Unigenes Identified after Trasncriptomics Analysis of Unfed Larvae

A total of 21,677,414 (∼2.1 Gb) Illumina 101 bp paired-end reads (207 bp average insert size) were subjected to analysis. After read assembly, 18,946 transcripts were obtained and annotated (Table S1). Transcripts were clustered by encoded proteins. If two transcripts were annotated as the same protein, then these transcripts were clustered together in the same protein cluster. We considered each set of transcripts annotated by the same protein as a unigene to identify transcripts from the same locus/gene. This approach identified a set of 3,808 unigenes with 1,231±286 (Ave±S.E) estimated counts per unigene (Table S1).

**Figure 1:**
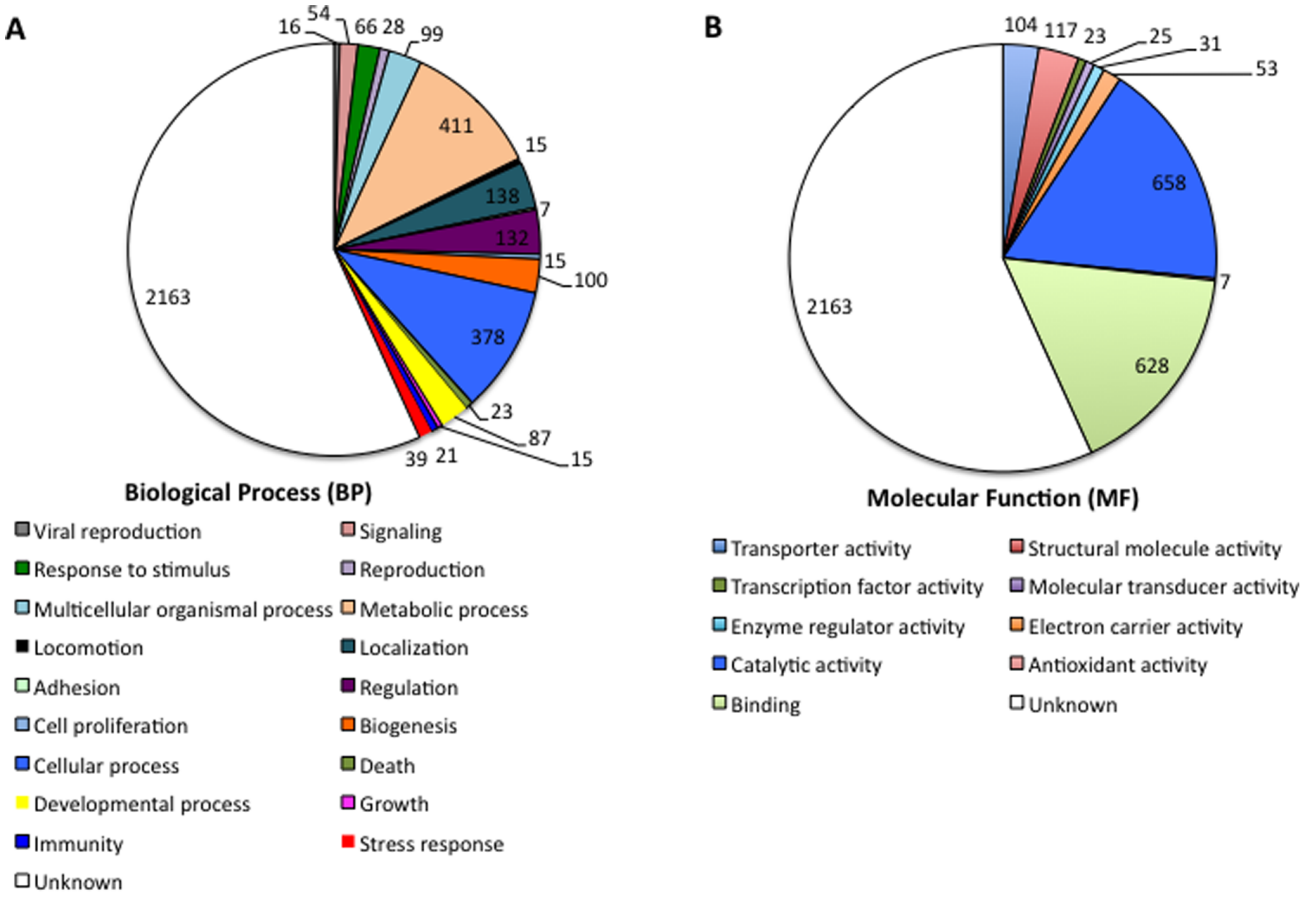
Transcriptomics of *D. reticulatus* unfed larvae. (A) Transcripts identified in *D. reticulatus* unfed larvae were functionally annotated and grouped according to the biological process of the encoded proteins. The number of proteins on each category is shown. (B) Transcripts identified in *D. reticulatus* unfed larvae were functionally annotated and grouped according to the molecular function of the encoded proteins. The number of proteins on each category is shown.

**Figure 2:**
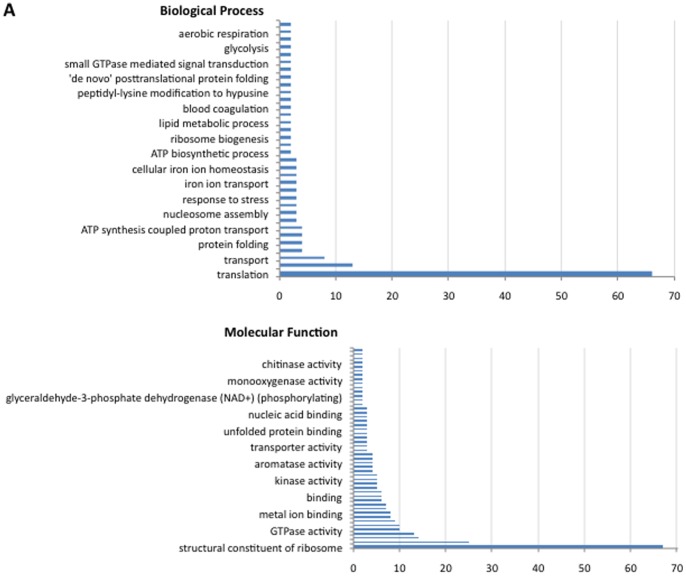
Five hundred most highly represented genes. (A) The 500 more represented unigenes (protein clusters) identified in *D. reticulatus* unfed larvae were functionally annotated and grouped according to the biological process of the encoded proteins. The number of proteins on each category is shown. (B) The 500 more represented unigenes (protein clusters) identified in *D. reticulatus* unfed larvae were functionally annotated and grouped according to the molecular function of the encoded proteins. The number of proteins on each category is shown.

The analysis of Biological Process (BP) and Molecular Function (MF) gene ontology (GO) showed that the most represented BPs corresponded to unknown process (N = 2,163; 57%), metabolic process (N = 411; 11%) and cellular process (N = 378; 10%) (Fig. 1A) while proteins with unknown function (N = 2,163; 57%), catalytic activity (N = 658; 17%) and binding activity (N = 628; 16%) were the most represented MFs (Fig. 1B). A closer analysis of the most expressed genes showed that translation and structural constituent of the ribosome were the most represented BP and MF in *D. reticulatus* unfed larvae, respectively (Figs. 2A and 2B). These genes encoded for 80S ribosomal proteins (Table 1). With the exception of yeast, which lacks L28e, eukaryotic cytoplasmic 80S ribosomes contain the same set of 80 core ribosomal proteins [7]. Thus, the transcripts identified in *D. reticulatus* larvae encoded for 72% (34/47) and 73% (24/33) of the large and small subunit 80S proteins, respectively (Table 1), representing a high coverage for ribosomal proteins. These results showed that metabolic and cellular processes involved in protein synthesis were the most active in *D. reticulatus* unfed larvae (Figs. 1A, 1B, 2A, 2B), suggesting that tick metabolism is highly active during this life stage.

**Table 1:** *D. reticulatus* 80S ribosome transcriptomics and proteomics data.

Uniprot ID	Protein name	New name
Large subunit proteins		
A5I8N9	Ribosomal protein L23	L23
A6N9V5	Ribosomal protein L21	L21e
A6N9Z6	60s ribosomal protein L10	L16
A9QQ32	60S ribosomal protein L30	L30e
A9QQ40	60s ribosomal protein L14	L14e
A9QQD0	60S ribosomal protein l27a	L15
B5M6D2	60S ribosomal protein L28	L28e
B5M6D3	60S ribosomal protein L13a	L13
B7P169	60S ribosomal protein L6	L6e
B7P3X5	Ribosomal protein L1	L1
B7PCP6	Ribosomal protein L35, putative	L29
B7PFS8	60S ribosomal protein L5, putative	L18
B7PSE0	Ribosomal protein L4, putative	L4
B7Q1E7	60S ribosomal protein L24, putative	L24e
B7QKS6	Ribosomal protein L19	L19e
B7QLY8	Ribosomal protein L3, putative	L3
B7QMT2	Ribosomal protein L39, putative	L39e
B7SP55	Ribosomal protein L31	L31e
C9W1H1	Ribosomal protein L12	L11
C9W1J8	24 4.5 60S ribosomal protein L37	L37e
C9W1K2	60S ribosomal protein L14	L14
C9W1L7	60S ribosomal protein L9	L6
C9W1P0	Ribosomal protein L21	L21e
E2J6X9	Ribosomal protein L15 (Fragment)	L15e
E7D150	Ribosomal protein L32 isoform B (Fragment)	L32e
P48159	60S ribosomal protein L23 (L17A)	L14e
P49632	60S ribosomal protein L40	L40e
Q4PM12	60S ribosomal protein L36	L36e
Q4PM17	Ribosomal protein L35a	L33e
Q4PM18	Ribosomal protein L34	L34e
Q4PM25	Ribosomal protein L37	L37e
Q4PM27	Ribosomal protein L11	L5
Q4PM37	Ribosomal protein L7-like	L30
Q4PM43	Ribosomal protein L15	L15e
Q4PM81	60S ribosomal protein L44	L44e
Q4PMD1	60S ribosomal protein L38	L38e
Q09JS1	Ribosomal protein LP1	P1
Small subunits proteins		
A6N9R2	Ribosomal protein S18	S13
A6N9Y3	40S ribosomal protein S27	S27e
A9QQ37	40s ribosomal protein S15	S19
A9QQ87	40S ribosomal protein S7	S7e
A9QQA8	40S ribosomal protein S5	S7
B7P2T4	Ribosomal protein S17, putative	S17e
B7QLZ5	40S ribosomal protein S9, putative	S4
C9W1H8	40S ribosomal protein S14	S11
C9W1M4	40S ribosomal protein S5	S7
E2J6R1	40S ribosomal protein S2/30S ribosomal protein S5	S5
E2J6W6	40S ribosomal protein SA (P40)/laminin receptor 1 (Fragment)	S2
E7D134	Ribosomal protein S16 (Fragment)	S9
E7D1C2	Putative ribosomal protein SA (Fragment)	S2
E7D1D5	40S ribosomal protein S8 (Fragment)	S8e
F0J926	40S ribosomal protein S3a (Fragment)	S1e
P48149	40S ribosomal protein S15A	S8
Q09JW5	Ubiquitin/40S ribosomal protein S27a fusion protein	S31e
Q4PM11	40S ribosomal protein S13	S15
Q4PM13	40S ribosomal protein S11	S17
Q4PM31	40S ribosomal protein S3a	S1e
Q4PM47	40S ribosomal protein S29	S14
Q4PM64	40S ribosomal protein S21	S21e
Q4PM65	40S ribosomal protein S12	S12e
Q4PM67	Ribosomal protein S16	S9
Q86G63	40S ribosomal protein S11	S17
Q4PMB3	40S ribosomal protein S4	S4e
Q4PMC1	40S ribosomal protein S8	S8e
Q4PMC2	Ribosomal protein S20	S10
Q86FP7	40S ribosomal protein S23	S12

Unigenes corresponding to 80S ribosomal proteins are shown. New names refer to current nomenclature for *D. melanogaster* [7]. The 80S ribosomal proteins (new/old name) L2/L8, L8e/L7A, L10/LP0, L13e/L13, L18e/L18, L20e/L18A, L22e/L22, L24/L26, L27e/L27, L29e/L29, L41e/L41, L43e/L37A, P2/LP2, RACK1/RACK1, S3/S3, S6e/S6, S10e/S10, S19e/S19, S24e/S24, S25e/S25, S26e/S26, S28e/S28, and S30e/S30 were not identified.

### *D. reticulatus* Proteins Identified after Proteomics Analysis of Unfed Larvae

Proteomics analysis was replicated using two different experimental approaches to increase the probability of identifying low abundant proteins such as those involved in stress response. In both approached, mass spectra were searched against Ixodida protein database. The first approach used protein concentration and resulted in the identification of 74 proteins while the second approach analyzed proteins separated by SDS-PAGE and resulted in 239 proteins identified (Table S2), suggesting that for non-quantitative analysis protein fractionation provides better resolution. Of 74 proteins identified with the first approach, 26 (35%) were identified by both methods.

A recently described technique named proteomics informed by transcriptomics (PIT) [8] was used against data generated by the first proteomics approach to validate this method in ticks. This approach uses a database created from transcriptomics data to search mass spectra and has been reported to increase the number of identified proteins [8]. PIT approach resulted in 104 proteins identified in unfed tick larvae (Table S2), representing a 40% increase with respect to the search against Ixodida protein database. The analysis of de novo sequences increased the number of identified proteins using both approaches for proteomics data analysis (Table S2). However, while de novo protein sequences represented 4% (N = 3) of the identified proteins searching against Ixodida protein database, the number of identified proteins increased in 47% (N = 49) using PIT (Table S2). These results support the use of PIT for the analysis of proteomics data, particularly for organisms such as *D. reticulatus* with little sequence information available.

**Figure 3:**
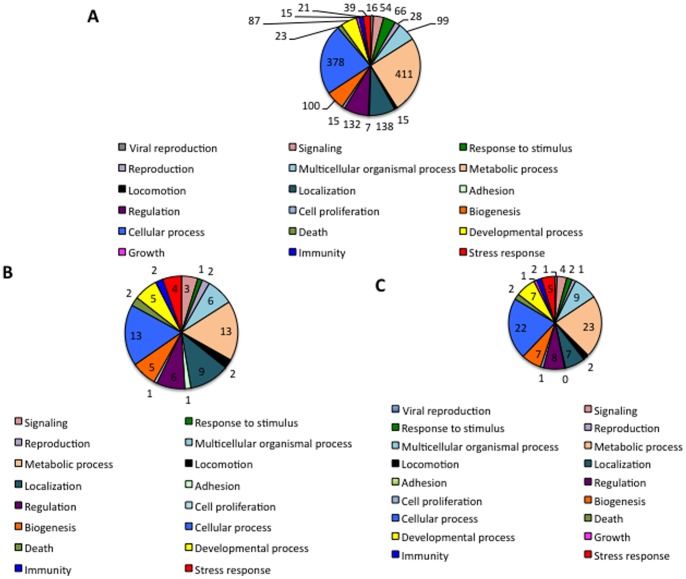
Biological processes identified in *D. reticulatus* unfed larvae. (A) Transcripts identified in *D. reticulatus* unfed larvae were functionally annotated and grouped according to the biological process of the encoded proteins after removing transcripts with unknown function. (B) Proteins identified in *D. reticulatus* unfed larvae after searching against Ixodida database were functionally annotated and grouped according to their biological process. (C) Proteins identified in *D. reticulatus* unfed larvae after searching against transcripts database (PIT) were functionally annotated and grouped according to their biological process. The number of proteins on each category is shown.

**Figure 4:**
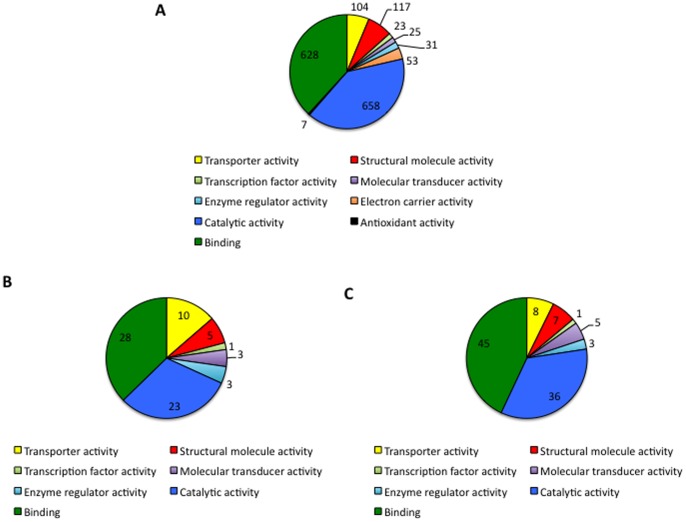
Molecular functions indentified in *D. reticulatus* unfed larvae. (A) Transcripts identified in *D. reticulatus* unfed larvae were functionally annotated and grouped according to the molecular function of the encoded proteins after removing transcripts with unknown function. (B) Proteins identified in *D. reticulatus* unfed larvae after searching against Ixodida database were functionally annotated and grouped according to their molecular function. (C) Proteins identified in *D. reticulatus* unfed larvae after searching against transcripts database (PIT) were functionally annotated and grouped according to their molecular function. The number of proteins on each category is shown.

After removing proteins with unknown BP and MF, transcriptomics and proteomics data correlated well with respect to the most represented BPs (Figs. 3A–3C) and MFs (Figs. 4A–4C). These results were similar for both proteomics approaches, showing a good correlation in the proteomics analysis and providing additional support for the identified mechanisms active in *D. reticulatus* unfed larvae.

### *Rickettsia* sp. Identified in *D. reticulatus* Unfed Larvae

Although these ticks were obtained from a colony considered to be free of tick-borne rickettsiae, some reads matching *Rickettsia* spp. were identified in *D. reticulatus* unfed larvae resulting in 16 unigenes (Table S3). These transcripts were probably wrongly annotated as *I. scapularis* proteins in Uniprot when they are likely *Rickettsia* proteins. In these cases, the Uniref representative protein of the cluster to which belongs the *I. scapularis* protein is a *Rickecttsia* protein and the rest of the members of the Uniref90 cluster are also from *Rickettsia*. Proteomics analysis corroborated the presence of *Rickettsia* proteins in *D. reticulatus* unfed larvae with the identification of 14 proteins searching against Rickettsiae database (Table S3).

The *Rickettsia* sp. identified in unfed larvae could be a commensal bacterium that has been described in *Dermacentor* and other tick species, but not in *D. reticulatus* [9]–[12] or a pathogen [13]. The *Rickettsia* proteins identified in *D. reticulatus* unfed larvae are highly conserved among *Rickettsia* spp. and thus not suitable to characterize these organisms at the species level.

To gain further information on this *Rickettsia* sp., the PCR amplification and sequencing of gene markers that have been previously used for species classification was conducted [14]–[16]. The results showed >99% pairwise nucleotide sequence identity to *Rickettsia* sp. sequences, especially to *R. raoultii* (Table 2). As previously shown [15], the *in silico Pst*I and *Rsa*I restriction analysis of *ompA* sequences was highly informative and corroborated that the *Rickettsia* sp. identified in this study is similar to *R. raoultii*. These results suggested, as in previous studies in tick cell culture [17], that the *Rickettsia* sp. identified in *D. reticulatus* unfed larvae is closely related to the tick-borne pathogen, *R. raoultii*. However, until this *Rickettsia* sp. is fully characterized, we cannot exclude the possibility of a symbiont closely related to *R. raoultii*. These results suggested that the pathogen could be an additional stress factor in *D. reticulatus* unfed larvae, which correlated with the activation of immune response in these ticks (Figs. 1A, 3A and 3C). *Rickettsia* sequences were deposited in the GenBank with accession numbers [GenBank: KF478838, KF478839].

**Table 2:** Sequence identity of the *Rickettsia* sp. identified in *D. reticulatus* unfed larvae.

Gene marker	*Rickettsia* sp. (Genbank accession no.)	Sequence identity
*atpA*	*R. raoultii* (KC428000)	99%
*dnaK*	*R. sibirica* subsp. *mongolitimonae* (KC428015)*R. massiliae* (KC428014)*R. slovaca* (CP003375)	100%
*16S rDNA*	*R. raoultii* (EU036982)*Rickettsia* sp. RpA4 (AF120026)	100%
*ompB*	*R. raoultii* (DQ365797)Uncultured *Rickettsia* sp. clone R2012 (JQ320341)	100%
*ompA*	*R. raoultii* (HM161789)	100%
*recA*	*R. raoultii* (KC428038)*R. massiliae* (GQ144452)	99%

### Stress Response in *D. reticulatus* Unfed Larvae

The results showed that metabolic processes and translation in particular were highly represented at the transcriptional level by genes encoding 80S ribosomal proteins in *D. reticulatus* unfed larvae (Table 1). Stress regulates ribosomal protein expression in other organisms, but no information is available in ticks [18]–[21]. Furthermore, a growing body of evidence suggests that the ribosome serves as a hub for co-translational folding, chaperone interaction, degradation, and stress response [22]. These results suggested a connection between transcription of ribosomal protein genes and stress response in ticks that deserves further investigation.

**Figure 5:**
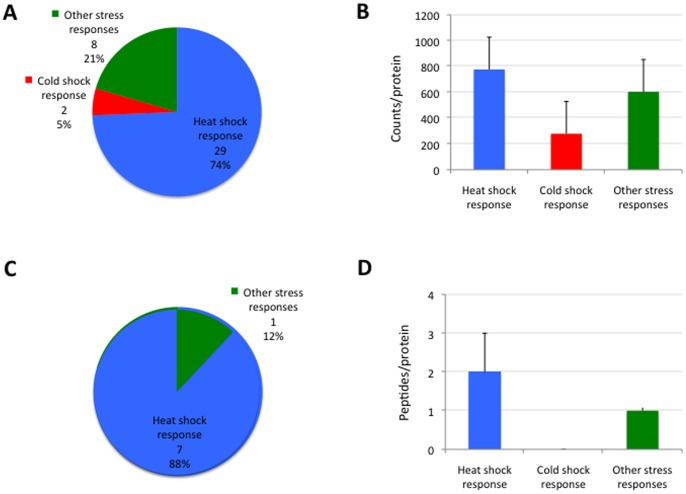
Stress response in *D. reticulatus* unfed larvae. (A) Stress response transcripts identified in *D. reticulatus* unfed larvae were grouped according to the function of their encoded protein. The number of proteins and percent in each category is shown. (B) Number of counts per protein (Ave+S.E.) in stress response proteins identified by transcriptomics analysis in *D. reticulatus* unfed larvae. (C) Stress response proteins identified in *D. reticulatus* unfed larvae were grouped according to the function of their encoded protein. The number of proteins and percent in each category is shown. (D) Number of peptides per protein (Ave+S.D.) in stress response proteins identified by proteomics analysis in *D. reticulatus* unfed larvae.

Transcripts and proteins mapped to stress response BP in *D. reticulatus* unfed larvae were selected for further analysis. Transcriptomics results showed that heat shock, cold shock and other stress responses were active in unfed larvae, represented by 39 unigenes (1% of all identified unigenes) and 27,937 counts (Table 3). Of them, the most represented functions corresponded to heat shock response (Figs. 5A and 5B). In general, protein identification has a lower resolution when compared to transcriptomics, a limitation that is more evident when working with species such as *D. reticulatus* for which sequence information is very scarce in the databases [23]. The search of MS data against the Ixodida database resulted in 8 stress response proteins identified (Table 4). However, when a database of transcripts identified as encoding for stress response proteins was generated and used for targeted PIT analysis, the results showed that 16 new stress response proteins were identified (Table 4). Additionally, while only 1% of the unigenes corresponded to stress response proteins, over 7% of the identified proteins were involved in this BP, supporting that stress response is active in tick unfed larvae. Furthermore, in agreement with transcriptomics data, the most represented function corresponded to heat shock response (Figs. 5C and 5D).

**Table 3:** Tick stress response proteins identified in *D. reticulatus* unfed larvae after transcriptomics analysis.

Uniprot ID	Counts perprotein	Protein name	Gene name	Organism
**Heat shock response proteins**				
B7PAR6	6894	HSP	ISCW017456	*Ixodes scapularis*
B7QI01	5601	HSP90	ISCW014265	*Ixodes scapularis*
L7M330	1830	Heat shock-related protein		*Rhipicephalus pulchellus*
E4W3Z2	1609	HSP70 protein 5		*Haemaphysalis longicornis*
L7LXP1	805	HSP90 co-chaperone p23		*Rhipicephalus pulchellus*
E2J6U8	652	Mitochondrial HSP60		*Hyalomma marginatum rufipes*
G3MSI6	608	HSP40		*Amblyomma maculatum*
L7M4B9	593	Heat shock-related protein		*Rhipicephalus pulchellus*
E0YPC0	577	Small HSP II		*Rhipicephalus annulatus*
I1ZDN9	455	HSP cognate 5	Hsc70-5	*Aeroglyphus robustus*
L7MCC0	435	HSP		*Rhipicephalus pulchellus*
F1CGQ9	334	HSP90	hsp90	*Panonychus citri*
B7P1Z8	301	HSP	ISCW016090	*Ixodes scapularis*
L7MFL0	257	Heat shock transcription factor		*Rhipicephalus pulchellus*
L7M6S1	227	Heat shock-related protein		*Rhipicephalus pulchellus*
G8Z375	220	HSP70-3		*Panonychus citri*
Q0V9A5	211	HSP70-1	hspa1l hspa1b	*Xenopus tropicalis*
B5M740	195	HSP90		*Amblyomma americanum*
J7G3V2	173	Heat shock cognate protein 70		*Latrodectus hesperus*
L7M1L7	137	Heat shock-related protein		*Rhipicephalus pulchellus*
B4YTU0	128	HSP70-3		*Tetranychus cinnabarinus*
L7LYK1	121	Heat shock transcription factor		*Rhipicephalus pulchellus*
F0J9M7	35	HSP9		*Amblyomma variegatum*
D8KWR5	33	HSP70		*Haemaphysalis longicornis*
B7P8Q5	33	HSP70	ISCW017192	*Ixodes scapularis*
L7M513	30	Putative ahsa1 c14orf3 hspc322: activatorof 90 kDa HSP atpase log 1		*Rhipicephalus pulchellus*
L7M6W4	21	HSP60		*Rhipicephalus pulchellus*
L7M597	16	HSP40		*Rhipicephalus pulchellus*
B7PRX5	14	Heat shock transcription factor	ISCW007739	*Ixodes scapularis*
**Cold shock response proteins**				
B7PD37	531	Translation initiation factor 2, alpha subunit	ISCW017360	*Ixodes scapularis*
L7MEM0	27	Putative cold shock domain protein		*Rhipicephalus pulchellus*
**Other stress response proteins**				
Q2XW15	2934	Glutathione peroxidase	PHGPX	*Rhipicephalus microplus*
L7M323	504	Putative nucleotide kinase/nuclearprotein involved oxidative stress response		*Rhipicephalus pulchellus*
B7QC85	459	Tumor rejection antigen, Gp96	ISCW022766	*Ixodes scapularis*
B7QG63	419	Glutathione peroxidase	ISCW022517	*Ixodes scapularis*
B7PUM7	232	Peroxinectin	ISCW007552	*Ixodes scapularis*
B7PP36	182	Peroxinectin	ISCW006862	*Ixodes scapularis*
P62140	64	Serine/threonine-protein phosphatasePP1-beta catalytic subunit	PPP1CB	*Homo sapiens*
L7M2W8	39	Putative bola bacterial stress-induced morphogen-related protein		*Rhipicephalus pulchellus*

**Table 4:** Tick stress response proteins identified in *D. reticulatus* unfed larvae after proteomics analysis.

Uniprot ID	Peptides perprotein	Protein name	Gene name	Organism
**Heat shock response proteins**				
B7QI01^a^^,^ b ^,^ c	2	HSP90	ISCW014265	*Ixodes scapularis*
B7PAR6^a^^,^ c	3	HSP	ISCW017456	*Ixodes scapularis*
E4W3Z2^a^^,^ c	4	HSP70		*Haemaphysalis longicornis*
B4YTU0^a^^,^ c	1	HSP70-3		*Tetranychus cinnabarinus*
F0J8P3^b^	4	HSP70		*Amblyomma variegatum*
L7MEG0^b^	3	HSP90		*Rhipicephalus pulchellus*
F0J9B6^b^	2	HSP		*Amblyomma variegatum*
B7PEN4^b^	2	HSP70	ISCW017754	*Ixodes scapularis*
G8Z375^c^	3	HSP70		*Panonychus citri*
L7M6W4^c^	2	HSP60		*Rhipicephalus pulchellus*
Q0V9A5^c^	2	HSP70		*Xenopus tropicalis*
L7M513^c^	2	HSP90 activator		*Rhipicephalus pulchellus*
L7M1L7^c^	2	Heat-shock related protein		*Rhipicephalus pulchellus*
G3MSI6^c^	2	HSP40		*Amblyomma maculatum*
G3MF42^b^	1	HSP20		*Amblyomma maculatum*
G3MNL6^b^	1	HSP20		*Amblyomma maculatum*
B7P8Q5^c^	1	HSP70	ISCW017192	*Ixodes scapularis*
E0YPC0^c^	1	Small HSP II		*Rhipicephalus annulatus*
B7QJZ5^b^	1	HSP	ISCW023475	*Ixodes scapularis*
**Cold shock response proteins**				
L7MEM0^c^	2	Putative cold shock domain protein		*Rhipicephalus pulchellus*
**Other stress response proteins**				
B7PUM7^c^	2	Peroxinectin	ISCW007552	*Ixodes scapularis*
P62140^c^	1	Serine/threonine-protein phosphatasePP1-beta catalytic subunit	PPP1CB	*Homo sapiens*
Q2XW15^c^	1	Glutathione peroxidase	PHGPX	*Rhipicephalus microplus*
B7QC85^a^^,^ c	1	Tumor rejection antigen, Gp96	ISCW022766	*Ixodes scapularis*

a Identified by PIT.

b Identified searching against Ixodida.

c Identified by targeted PIT.

**Figure 6:**
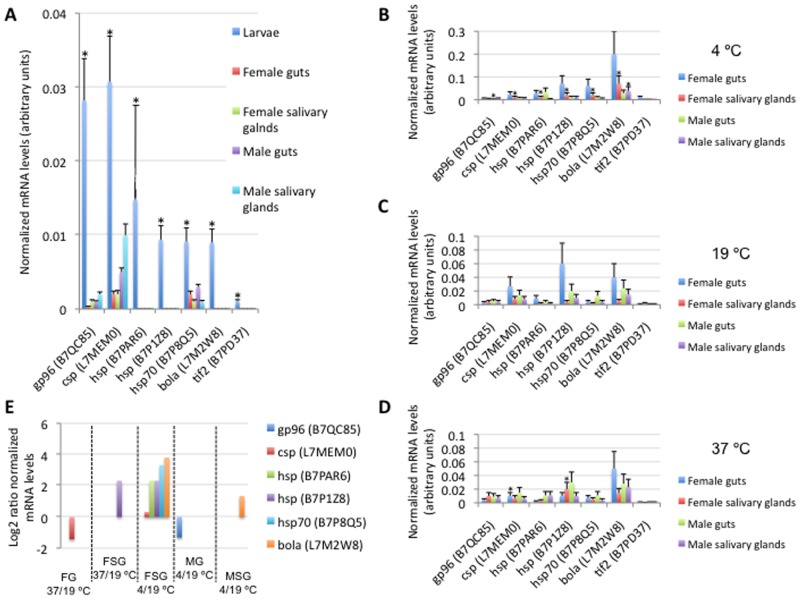
mRNA levels for selected genes encoding for stress response proteins. (A) The mRNA levels were characterized by real-time RT-PCR in *D. reticulatus* unfed larvae and adult female and male guts and salivary glands (N = 3), normalized against tick ribosomal protein S4 and shown as Ave+S.D. in arbitrary units. Normalized Ct values were compared between larvae and adult samples by Student's t-test with unequal variance (*P≤0.05). (B–D) The mRNA levels were characterized by real-time RT-PCR in *D. reticulatus* guts and salivary glands from adult female and male ticks incubated at 4, 19 and 37°C for 4.5 h prior to RNA extraction (N = 3), normalized against tick ribosomal protein S4 and shown as Ave+S.D. in arbitrary units. Normalized Ct values were compared between samples from ticks incubated at 4 or 37°C and 19°C by Student's t-test with unequal variance (*P≤0.05). (E) For genes with significant differences between samples from ticks incubated at 4°C or 37°C and 19°C, the log2 4/19°C or 37/19°C normalized Ct values ratio was calculated to show differential expression in response to temperature. Abbreviations: FG, female gusts; FSG, female salivary glands; MG, male guts; MSG, male salivary glands.

Some transcripts mapped to stress response BP were selected for the characterization of mRNA levels in *D. reticulatus* tick unfed larvae and guts and salivary glands from adult ticks incubated at 4, 37 or 19°C by real-time RT-PCR (Figs. 6A–6E). The results showed that all selected genes encoding for stress response proteins were more expressed in unfed larvae than in adult tissues, thus reinforcing the significance of this BP in *D. reticulatus* tick unfed larvae (Fig. 6A). In adult ticks, some genes were differentially expressed in response to temperature changes in guts or salivary glands (Figs. 6B–6E). The differential expression of selected genes encoding for stress response proteins was more evident in female salivary glands than in female guts and male tissues (Fig. 6E), suggesting differences between female and male ticks and between tissues in stress response to temperature changes. Additionally, at least for the genes characterized in this experiment, differential expression was more pronounced at 4°C than at 37°C (Fig. 6E), suggesting that *D. reticulatus* ticks respond differently to different temperatures. The sequences of *D. reticulatus* genes encoding for stress response proteins were deposited in the GenBank with accession numbers [GenBank: SRR950367; Bioproject: PRJNA214849].

Ticks are very sensitive to temperature and their life cycle is dependent on a complex combination of climate variables for development and survival [24]. In particular, *D. reticulatus* tick unfed larvae are exposed to extremely stressing conditions that affect their survival and development [5]. The heat-shock and other stress responses are a conserved reaction of cells and organisms to different stress conditions such as extreme temperatures, toxicity and pathogen infection [25]. Crucial to cell survival is the sensitivity of proteins and enzymes to heat inactivation and denaturation. Therefore, adaptive mechanisms exist that protect cells from the proteotoxic effects of stress. The heat-shock proteins and other stress response proteins protect cells and organisms from damage, providing higher levels of tolerance to environmental stress. Recent studies demonstrated that the stress response is activated in ticks and cultured tick cells in response to *Anaplasma* spp. infection and heat shock [26], [27]. These results showed that at high temperatures and during blood feeding, when *hsp20*, *hsp70* and *subolesin* are overexpressed, *Ixodes scapularis* ticks are protected from stress and pathogen infection and have a higher questing speed. Herein, genes encoding for stress response proteins were also differentially expressed in *D. reticulatus* in response to cold or heat shock. These results suggested a connection between tick stress response, questing behavior and pathogen infection [26], [27], which may be present also in *D. reticulatus* tick unfed larvae. Experiments characterizing the mRNA and protein levels of genes identified in this study in *D. reticulatus* ticks exposed to blood feeding and pathogen infection would add additional support to the importance of these proteins in tick stress response.

## Conclusions

The characterization of the transcriptome and proteome of *D. reticulatus* unfed larvae showed that stress response was active in this developmental stage. Although descriptive in its nature, these results showed that combination of transcriptomics and proteomics approaches provide strong support for the characterization of biologically relevant pathways in ticks. The activation of stress responses in *D. reticulatus* unfed larvae likely counteracts the negative effect of temperature and other stress conditions such as *Rickettsia* infection and favors tick adaptation to environmental conditions to increase tick survival. These results are relevant to understand how *D. reticulatus* ticks have evolved mechanisms to survive under stress conditions and suggest that these mechanisms are conserved across hard tick species. Targeting some of these proteins by vaccination may increase tick susceptibility to natural stress conditions, which in turn reduce tick survival and reproduction, thus reducing tick populations and vector capacity for tick-borne pathogens [28].

## Materials and Methods

### Experimental Design and Rationale

In this research, we completed the analysis of the transcripts and proteins present in *D. reticulatus* unfed larvae, which are described in Tables S1 and S2. This information, which was not available for this species, was then used to characterize stress response by focusing on the relevant genes and proteins. Individual variability, which certainly exists in ticks as in other organisms, was considered by pooling a large number of larvae for transcriptomics (N = 500) and proteomics (N = 200) studies. As in previous studies [29]–[31], we did not use biological replicates for RNA-Seq but the algorithm used to quantitate transcriptomics data allows the use of non-replicated samples [32]. Proteomics analysis, although also used for a non-comparative study that does not require replicates [31], was replicated using a different experimental approach to increase the probability of identifying low abundant proteins such as those involved in stress response. The statistical significance of reads and peptide assignments is supported by the application of eXpress and SEQUEST (FDR<0.01) algorithms described bellow for the analysis of transcriptomics and proteomics data, respectively.

### Ticks and Sample Preparation

*D. reticulatus* unfed larvae were obtained from a single female from a Dutch colony maintained at the Utrecht Centre for Tick-borne Diseases (UCTD), Department of Infectious Diseases and Immunology, Faculty of Veterinary Medicine, Utrecht University, Utrecht, The Netherlands. Total RNA and DNA were extracted from approximately 500 *D. reticulatus* larvae kept off-host for 7 days using the AllPrep DNA/RNA/Protein Mini Kit (Qiagen, Valencia, CA, USA) according to manufacturer instructions. RNA was purified with the RNeasy MinElute Cleanup Kit (Qiagen, Valencia, CA, USA) and characterized using the Agilent 2100 Bioanalyzer (Santa Clara, CA, USA) in order to evaluate the quality and integrity of RNA preparations. RNA concentration was determined using the Nanodrop ND-1000 (NanoDrop Technologies Wilmington, Delaware USA). For protein extraction, approximately 200 *D. reticulatus* larvae were pulverized in liquid nitrogen and homogenized with a glass homogenizer (20 strokes) in 4 ml buffer (0.25 M sucrose, 1 mM MgCl_2_, 10 mM Tris-HCl, pH 7.4) supplemented with 4% SDS and complete mini protease inhibitor cocktail (Roche, Basel, Switzerland). Sample was sonicated for 1 min in an ultrasonic cooled bath followed by 10 sec vortex. After 3 cycles of sonication-vortex, the homogenate was centrifuged at 20×g for 5 min at room temperature to remove cellular debris. The supernatant was collected and protein concentration was determined using the BCA Protein Assay (Thermo Scientific, San Jose, CA, USA) using BSA as standard.

*D. reticulatus* unfed female and male adults were obtained from a tick colony originally collected in southern Slovakia and maintained at the Biology Centre of the ASCR, Parasitology Institute, České Budějovice, Czech Republic.

### Transcriptomics Data Acquisition

The RNA purified from unfed tick larvae was used for library preparation using the TruSeq RNA sample preparation kit v.1 and the standard low throughput procedure (Illumina, San Diego, CA, USA). Briefly, 0.7 µg total RNA was used as starting material for library preparation. Messenger RNA was captured using poly-dT magnetic beads and purified polyA+ RNA was chemically fragmented and reverse-transcribed. Remaining RNA was enzymatically removed and the second strand generated following an end repair procedure and preparation of double-stranded cDNA for adaptor ligation. Adaptor oligonucleotides containing the signals for subsequent amplification and sequencing were ligated to both ends and the cDNA was washed using AMPure SPRI-based magnetic beads (Beckman Coulter, IZASA, Barcelona, Spain). Adaptors contained identifiers, which allow multiplexing in the sequencing run. An enrichment procedure based on PCR was then performed to ensure that all molecules in the library conserved the adapters at both ends. The number of PCR cycles was adjusted to 15. The final amplified library was checked again on a BioAnalyzer 2100 (Agilent, Santa Clara, CA, USA) and titrated by quantitative PCR using a reference standard to characterize molecules concentration in the library (12.44 nM). The library was denatured and seeded on the lane of the flowcell at a final concentration after re-naturalization of 10–14 pM. After binding, clusters were formed in the flowcell by local amplification using a Cluster Station apparatus (Illumina). Following sequencing primer annealing, flowcell was loaded into a GAIIx equipment (Illumina) to perform sequencing using the TruSeq® system (Illumina). The sample was run under a pair-end 2×100 bp protocol for de novo sequencing. After sequencing and quality filtering, reads were split according to their different identifiers and fastq files were generated to proceed to quality analysis and de novo transcript assembly and gene expression analysis.

### Bioinformatics for Transcriptomics Data

Sequence reads were trimmed at the error probability higher than 0.05 and assembled only when two members of the pair remained after filtering at trimming. Oases [33] was used for read assembly in the mode of single (not merged) assembly because results were better in this mode. A K value of 79 was chosen, which was very close to the total length of the read (∼100 bp) to avoid misassemblies since the higher the overlapping required the more accurate the transcript is. Final assembly was explored in detail using Tablet (http://bioinf.scri.ac.uk/tablet/download.shtml) [34].

Functional annotations were inferred by similarity to Uniprot reference proteins using Blast E values <10E-10. We selected a set of 34,095 reference proteins downloaded from Uniprot on March 7, 2013, including all proteins that were representative of Uniref90 clusters belonging to the taxonomic node Chelicerata, which are 8 levels above *D. reticulatus* taxon. In the Uniref90 clusters, each protein belongs to only one cluster with a 90% similarity to the representative protein for all members of the cluster. It provides a more homogeneous and uniform distance between reference proteins. Reference proteins were used for transcript clusterization to obtain a protein-centred analysis of gene expression that is more useful for functional analysis in a *de novo* transcriptome.

The eXpress algorithm was used for mapping reads to multiple targets to quantify gene expression levels [32]. The eXpress algorithm [32] for quantifying the abundances of the transcripts addresses multi-mapping based on an on-line expectation–maximization algorithm (online-EM) [35] that is used to estimate transcript abundances in multi-isoform genes and gene families, and that does not require a reference genome. The underlying model is based on previously described probabilistic models developed for RNA-seq and allows the use of parameters for fragment length distributions, errors in reads, and sequence-specific fragment bias [36]. The algorithm alternates between assigning fragments to targets with a probability according to abundance parameters (expectation step) and updating abundances to the maximum-likelihood solution on the basis of the expectation-step assignments (maximization step). At the beginning the abundances are set to a uniform initial value. Then, for the fragments that map to multiple sites, eXpress calculates probabilities for each site, considering previous estimates of target-sequence abundances. As fragments are processed, they are assigned increasing ‘mass’ to improve the estimation of abundance according to the assignment probability. Parameters for fragment-length (L) distribution, sequence bias and sequence read errors are updated and used in the next round of assignment. While relative abundance and count estimates are updated, uncertainties in assignment are propagated so that posterior count distributions can be estimated. The probabilistic model is described in detail in the online methods section in Roberts and Pachter [32].

eXpress was also used to analyze the read mapping results. The mapper tool used was Bowtie setting the mapping parameters following the eXpress recommendations. Bowtie is an ultrafast, memory-efficient short read aligner that indexes the reference with a Burrows-Wheeler index to have low memory requirements [37]. Bowtie indexes the reference genome using a scheme based on the Burrows-Wheeler transform (BWT) [38], that is a reversible permutation of the characters in a text developed for data compression and the Ferragina and Manzini (FM) index [39]. Bowtie adopts the exact-matching algorithm of Ferragina and Manzini for searching in the FM index but introduces a quality-aware backtracking algorithm that allows mismatches and 'double indexing', to avoid excessive backtracking.

The script used for mapping the reads to the transcripts with Bowtie and for the final quantification with eXpress (File S1) was performed using cloud computing (Amazon Web Services). The process took 100 minutes in an Amazon EC2 m2.4xlarge instance. This kind of instances has 8 virtual CPUs and 68.4 GiB of RAM.

### Proteomics Data Acquisition

#### Proteins concentrated by SDS-PAGE

The protein extract (150 µg) was precipitated following the methanol/chloroform procedure [40], resuspended in 100 µl Laemmli sample buffer and applied onto 1.2-cm wide wells on a 12% SDS-PAGE gel. The electrophoretic run was stopped as soon as the front entered 3 mm into the resolving gel, so that the whole proteome became concentrated in the stacking/resolving gel interface. The unseparated protein band was visualized by staining with GelCode Blue Stain Reagent (Thermo Scientific), excised, cut into 2×2 mm cubes and digested overnight at 37°C with 60 ng/µl sequencing grade trypsin (Promega, Madison, WI, USA) at 5∶1 protein:trypsin (w/w) ratio in 50 mM ammonium bicarbonate, pH 8.8 containing 10% (v/v) acetonitrile [41]. The resulting tryptic peptides from the gel band were extracted by 30 min-incubation in 12 mM ammonium bicarbonate, pH 8.8. Trifluoroacetic acid was added to a final concentration of 1% and the peptides were finally desalted onto OMIX Pipette tips C_18_ (Agilent Technologies, Santa Clara, CA, USA), dried-down and stored at −20°C until mass spectrometry analysis.

The desalted protein digest was resuspended in 0.1% formic acid and analyzed by RP-LC-MS/MS using an Easy-nLC II system coupled to an ion trap LCQ Fleet mass spectrometer (Thermo Scientific). The peptides were concentrated (on-line) by reverse phase chromatography using a 0.1×20 mm C18 RP precolumn (Thermo Scientific), and then separated using a 0.075×100 mm C18 RP column (Thermo Scientific) operating at 0.3 µl/min. Peptides were eluted using a 180-min gradient from 5 to 35% solvent B (Solvent A: 0,1% formic acid in water, solvent B: 0,1% formic acid in acetonitrile). ESI ionization was done using a Fused-silica PicoTip Emitter ID 10 µm (New Objective, Woburn, MA, USA) interface. Peptides were detected in survey scans from 400 to 1600 amu (1 µscan), followed by three data dependent MS/MS scans (Top 3), using an isolation width of 2 mass-to-charge ratio units, normalized collision energy of 35%, and dynamic exclusion applied during 30 sec periods.

#### Proteins separated by SDS-PAGE

The protein extract (150 µg) was precipitated following the methanol/chloroform procedure [40], resuspended in 100 µl Laemmli sample buffer and applied onto 1.2-cm wide wells on a 12% SDS-PAGE gel. The protein bands were visualized by staining with GelCode Blue Stain Reagent (Thermo Scientific) and sliced each gel lane into 25 slices as previously described [42]. Protein digestion and RP-LC-MS/MS analysis was performed as described before for proteins concentrated by SDS-PAGE.

### Bioinformatics for Proteomics Data

The MS/MS raw files were searched against Ixodida (40,849 entries in June 2013) and Rickettsieae (58,899 entries in June 2013) Uniprot databases and against a database created from transcriptomics data (PIT) [8] using the SEQUEST algorithm (Proteome Discoverer 1.3, Thermo Scientific) with the following constraints: tryptic cleavage after Arg and Lys, up to two missed cleavage sites, and tolerances of 1 Da for precursor ions and 0.8 Da for MS/MS fragment ions and the searches were performed allowing optional Met oxidation and Cys carbamidomethylation. For peptide validation, the Percolator node present in the Proteome Discoverer 1.3 software was used. Percolator is a machine-learning supplement of the Sequest algorithm that uses a decoy database search strategy to learn to distinguish between correct and incorrect peptide identifications increasing the sensitivity and specificity of peptide identification [43], [44]. The filtering criteria applied in this case are based on the q-value generated by Percolator that is defined as the minimal false discovery rate at which the identification is deemed correct [43]. These q-values are estimated using the distribution of scores from the decoy database search. A false discovery rate (FDR) <0.01 was considered as condition for successful peptide assignments, including only peptides with q-values ≤0.01 and delta Cn >0.05. *De novo* peptide sequencing was conducted with Peaks Studio 6.0 software (Bioinformatics Solutions Inc., Waterloo, ON Canada).

### Gene and Protein Ontology Assignments

Functional data for each protein were obtained from Uniprot and included GO annotations, EC number and Interpro motifs. Assignment of GO terms to identified proteins was done by Blast2GO software (version 2.6.6; http://www.blast2go.org/) in three main steps: blasting to find homologous sequences, mapping to collect GO-terms associated to blast hits and annotation to assign functional terms to query sequences from the pool of GO terms collected in the mapping step [45]. Sequence data of identified proteins were uploaded as FASTA file to the Blast2GO software and the function assignment was based on GO database. The blast step was performed against NCBI public databases through blastp. Other parameters were kept at default values: e-value threshold of 1e-3, recovery of 20 hits per sequence, minimal alignment length (hsp filter) 33 (to avoid hits with matching region smaller than 100 nucleotides) and Blast mode was set to QBlast-NCBI. Configuration for annotation was an e-value-Hit-filter of 1.0E-6, annotation cut off of 55 and GO weight of 5. For visualizing the functional information (GO categories: Molecular Function and Biological process), the analysis tool of the Blast2GO software was used.

The GO analysis for the 500 more represented unigenes was based on the GO annotations included in the Uniprot entry of the representative protein of each cluster. The GO analysis was done using Bio4j Go Tools developed by Era7 Bioinformatics and available at http://gotools.bio4j.com:8080/Bio4jTestServer/Bio4jGoToolsWeb.html. Bio4j Go Tools is a set of GO related Web Services using the open source graph bioinformatics platform Bio4j as back-end. Bio4j is a graph-based database including most data available in UniProt KB (SwissProt+Trembl), Gene Ontology (GO), UniRef (50,90,100), RefSeq, NCBI taxonomy, and Expasy Enzyme (http://bio4j.com/). Specifically designed java programs were used for the generation of the GO frequency chart data.

### Effect of Temperature on Gene Expression

Three groups of 9 *D. reticulatus* unfed female or male adults each were incubated for 4.5 h at 4°C, 19°C or 37°C and 20% relative humidity. After incubation, ticks were dissected and salivary glands and guts were separated, pooled in groups of three (3 groups for each temperature and sex) and immediately stored in TriReagent (Sigma, St. Louis, MO, USA) for RNA extraction.

### Analysis of mRNA Levels by Real-time RT-PCR

For real-time RT-PCR, tick larvae (three pools of 100 larvae each), unfed female and male adult ticks (3 ticks each) and guts and salivary glands from unfed female and adult adults incubated at different temperatures (3 groups for each temperature and sex) were used for RNA extraction using TriReagent (Sigma, St. Louis, MO, USA) following manufacturer’s recommendations. Real-time RT-PCR was performed on tick RNA samples (5 ng) with gene specific primers (20 pmol each) and conditions (Table 5) using the iScript One-Step RT-PCR Kit with SYBR Green and the iQ5 thermal cycler (Bio-Rad, Hercules, CA, USA) following manufacturer's recommendations. A dissociation curve was run at the end of the reaction to ensure that only one amplicon was formed and that the amplicons denatured consistently in the same temperature range for every sample [46]. The mRNA levels were normalized against tick ribosomal protein S4 using the genNorm method (ddCT method as implemented by Bio-Rad iQ5 Standard Edition, Version 2.0) [47], [48]. Normalized Ct vales were compared between larvae and adult samples and between samples from ticks incubated at 4 or 37°C and 19°C by Student's t-test with unequal variance (P = 0.05).

**Table 5:** Primer sequences used for real-time RT-PCR.

Gene (Uniprot ID)	Forward and reverse primers (5′-3′)	PCR annealing temperature
hsp (B7PAR6)	GACAAGGGCCGTCTGACAAA CGACTTGATAGCCTCCTCCT	(a)
hsp (B7P1Z8)	TTGAGGAGAAGCAGCACTGG GACGACTTCGGCTGTTGTTC	(b)
hsp70 (B7P8Q5)	TCGATATCCACCTCGTCCGT GCAGTAAGGAAGGGCAGGTT	(a)
Translation initiation factor 2 (*tif2*), alpha subunit (B7PD37)	CACTGATGCGTTGGCGAAAA CCGGACACTTCCCTGTTCTC	(a)
Putative cold shock domain protein, *csp* (L7MEM0)	CACTACAGCCAGTTCTCGGG CACCTCATCGCTAAGCACCT	(b)
Tumor rejection antigen, *gp96* (B7QC85)	CGGCTGTTGAAGAAGGGCTA CCCTCGTCAACCTTGAGACC	(b)
Putative *bola* bacterial stress-induced morphogen-related protein (L7M2W8)	TGAGCTGGAGGACGTTTCAG CATTCACCAGACGATGCTGC	(a)
Ribosomal protein S4, *rpS4* (DQ066214)	GGTGAAGAAGATTGTCAAGCAGAG TGAAGCCAGCAGGGTAGTTTG	(b)

PCR conditions: 40 cycles of 30 sec denaturation at 95°C, 30 sec annealing at (a) 55°C or (b) 60°C and 1 min extension at 72°C.

### PCR and Sequence Analysis of *Rickettsia* Amplicons

*Rickettsia* sp. DNA was characterized by PCR, cloning and sequence analysis of the amplicons. At least three clones were sequenced for each amplicon. Genes targeted by PCR included fragments of ATP synthase alpha subunit (*atpA*), heat-shock protein 70 (*dnaK*), outer membrane protein A (*ompA*), outer membrane protein B (*ompB*), 16S rRNA, and *recA* [14]–[16]. Nucleotide sequence identity to reference strains and *in silico Pst*I and *Rsa*I restriction analysis of *ompA* sequences was used to characterize *Rickettsia* sp. [15], [16].

## Supporting Information

Table S1 **Tick transcripts and encoded proteins identified in transcriptomics analysis of** ***D. reticulatus*** **unfed larvae.**
(XLSX)

Table S2 **Tick proteins identified in proteomics analysis of** ***D. reticulatus*** **unfed larvae.**
(XLSX)

Table S3 ***Rickettsia*** **sp. transcripts and proteins identified in** ***D. reticulatus*** **unfed larvae.**
(XLSX)

File S1 **Script used for mapping the reads to the transcripts with Bowtie and for the final quantification with eXpress.**
(DOCX)
