# Supplementary material for: A Systems Biology Approach to the Characterization of Stress Response in Dermacentor reticulatus Tick Unfed Larvae
Source: PLoS One. 2014 Feb 21;9(2):e89564. doi: 10.1371/journal.pone.0089564 (PMC3931811; doi:10.1371/journal.pone.0089564)
Supplement: File S1 — Script used for mapping the reads to the transcripts with Bowtie and for the final quantification with eXpress. (DOCX) [file pone.0089564.s004.docx]

#!/bin/sh

sample=LARVAE

reads1=${sample}.trimmed.Q30.noNs.good_1.fastq

reads2=${sample}.trimmed.Q30.noNs.good_2.fastq

echo "438"

tar -xvf LARVAE.processed-reads.tgz

# build the bowtie index

bowtie-build --offrate 1 LARVAE-assembly-k-79-transcripts.fa LARVAE-assembly-k-79-transcripts.fa

# map the reads

bowtie -aS -X 438 -p 8 --offrate 1 LARVAE-assembly-k-79-transcripts.fa -1 ./${reads1} -2 ./${reads2} | samtools view -Sb - > ./LARVAE-mapped-reads.bam

# running express

express ./LARVAE-assembly-k-79-transcripts.fa ./LARVAE-mapped-reads.bam -o ./output/

# get sort bam, bai and stats files

samtools sort ./LARVAE-mapped-reads.bam ./LARVAE-mapped-reads-sorted

samtools index ./LARVAE-mapped-reads-sorted.bam

samtools flagstat ./LARVAE-mapped-reads-sorted.bam > ./LARVAE-mapping-stats.txt
